# Impact of Highly Active Antiretroviral Therapy (HAART) on HIV-Related Cardiomyopathy: A Retrospective Study

**DOI:** 10.7759/cureus.103175

**Published:** 2026-02-07

**Authors:** Shamaiza Waqas, Luxhman Gunaseelan, Khurram Arshad, Majid Hanna, Waqas Abid, Ben Collins-Hamel, Steven Kehres

**Affiliations:** 1 Internal Medicine, Henry Ford Warren Hospital, Warren, USA; 2 Internal Medicine, Corewell Health Dearborn Hospital, Dearborn, USA; 3 Cardiology, Corewell Health Dearborn Hospital, Dearborn, USA; 4 Interventional Radiology, Henry Ford Warren Hospital, Warren, USA

**Keywords:** aging, cardiovascular outcomes, echocardiography, highly active antiretroviral therapy, hiv-associated cardiomyopathy, human immunodeficiency virus, left ventricular ejection fraction

## Abstract

Highly active antiretroviral therapy (HAART) has significantly improved outcomes in patients with human immunodeficiency virus (HIV) and may provide cardioprotective effects; however, its long-term impact on HIV-associated cardiomyopathy remains uncertain. Although short-term benefits have been reported, potential cardiovascular toxicity, particularly from protease inhibitors, warrants further investigation. This retrospective study evaluated HIV-infected patients with cardiomyopathy treated at five community hospitals in Michigan from 2015 to the present, examining changes in left ventricular ejection fraction (EF) following HAART therapy. Baseline EF measurements were obtained at or near the initiation of HAART, when available, although the exact timing varied across patients. EF changes were categorized as increased, unchanged, or decreased using a <10% threshold, consistent with the known interobserver variability of echocardiographic EF measurement. In this cohort, HAART exposure was not uniform, as treatment regimens, durations of therapy, and viral suppression statuses varied due to the retrospective nature of the study. Due to the limited availability of uniformly collected covariates, multivariable adjustment was not feasible, and the high exclusion rate resulting from missing treatment or follow-up imaging data introduces potential selection bias.

Of 125 patients identified, 50 met the inclusion criteria, while the remaining 75 were excluded due to incomplete data. Among the included patients, 44% (n = 22) demonstrated a decrease in EF, 28% (n = 14) showed an increase, and 28% (n = 14) had no significant change. Patients with decreased EF were older than those with increased EF (55.5 ± 10.3 vs. 47.9 ± 10.5 years; p = 0.04). When patients with decreased and unchanged EF were combined, age remained significantly higher compared with the increased EF group (p = 0.03), suggesting age as an important determinant of EF trajectory during HAART therapy. Gender and ethnic distributions did not differ significantly across EF groups. Males comprised 59% of the decreased EF group and 78.6% of both the unchanged and increased EF groups (p = 0.32). African American patients represented 77.3%, 63.4%, and 85.7% of the decreased, unchanged, and increased EF groups, respectively (p = 0.36). Alcohol use and chronic kidney disease were not significantly associated with EF changes (p = 0.84 and p = 0.43).

These findings identify age as a key factor influencing left ventricular EF changes in patients with HIV-associated cardiomyopathy receiving HAART. The absence of a control group, heterogeneity in HAART exposure, and incomplete comorbidity data limit causal interpretation. Larger controlled studies are needed to clarify the long-term cardiac effects of HAART and to determine how patient-level factors modify EF trajectories over time.

## Introduction

The introduction of highly active antiretroviral therapy (HAART) has altered the natural history of human immunodeficiency virus (HIV) infection, transforming it from a fatal illness into a chronic disease with near-normal life expectancy for many patients [1,2]. As survival has improved, non-AIDS-related conditions now account for a growing proportion of morbidity and mortality in people living with HIV, with cardiovascular disease emerging as a leading contributor [3,4].

Prior to the widespread use of HAART, HIV-associated cardiomyopathy was a well-recognized complication, typically presenting as dilated cardiomyopathy with reduced systolic function and poor clinical outcomes [5,6]. Proposed mechanisms included direct viral myocardial injury, immune-mediated inflammation, opportunistic infections, and nutritional deficiencies [6,7]. Several observational studies demonstrated improvement in left ventricular function following initiation of antiretroviral therapy, suggesting that viral suppression and immune reconstitution may confer cardioprotective effects [8,9].

Despite these early benefits, long-term exposure to antiretroviral therapy has raised concerns regarding cardiovascular toxicity. Protease inhibitors and certain nucleoside reverse transcriptase inhibitors have been associated with metabolic abnormalities, endothelial dysfunction, and accelerated atherosclerosis, all of which may contribute to adverse cardiovascular outcomes [10,11]. However, prior research has generally examined specific drug classes or short- to intermediate-term cardiac effects, leaving uncertainty about how HAART regimens affect myocardial function over longer periods. In addition, chronic immune activation and persistent low-grade inflammation persist even in virologically suppressed patients and may promote myocardial fibrosis and ventricular remodeling over time [12,13]. Thus, the net long-term effect of HAART on cardiac structure and function remains poorly defined, particularly with respect to left ventricular ejection fraction (EF) trajectories.

Age, sex, and race are important modifiers of cardiovascular risk in both the general population and among individuals with HIV infection. African American males are disproportionately affected by HIV and experience higher rates of cardiovascular disease and heart failure compared with other demographic groups [14-16]. Aging has also been associated with increased myocardial fibrosis, heightened inflammatory signaling, and a greater burden of comorbid cardiovascular risk factors, all of which may influence ventricular remodeling in the setting of chronic HIV infection. However, prior studies have not clearly established whether age modifies long-term EF changes among HAART-treated individuals or whether EF trajectories differ meaningfully across demographic groups. Accordingly, the present study sought to evaluate longitudinal changes in left ventricular EF among HIV-infected patients with cardiomyopathy receiving HAART and to identify demographic and clinical factors associated with EF change, with a particular focus on age-related effects and implications for patient outcomes.

The primary objective of this study was to evaluate longitudinal changes in left ventricular EF among HIV-infected patients with cardiomyopathy receiving HAART. The secondary objective was to determine whether demographic or clinical characteristics, particularly age, were associated with EF trajectory. This analysis examined HAART exposure at the regimen-class level and did not evaluate outcomes according to specific antiretroviral drug classes.

## Materials and methods

This retrospective study was conducted at five Ascension Southeast Michigan hospitals and included adult patients with HIV and a documented diagnosis of cardiomyopathy. Data were collected from 2015 to 2023. Patients were identified using the Ascension Data Warehouse, which contains billing and diagnostic records from all clinical encounters within the health system, through ICD-9 and ICD-10 codes for HIV and cardiomyopathy. Cardiomyopathy was defined based on clinical documentation and echocardiographic evidence of left ventricular systolic dysfunction; exclusions for ischemic versus nonischemic etiology could not be uniformly applied due to variable documentation across records. After identification, clinical charts were reviewed to obtain demographic information, antiretroviral therapy history, echocardiographic reports, and longitudinal follow-up information.

Patients were eligible for inclusion if they were 18-65 years of age, had a confirmed diagnosis of HIV infection and cardiomyopathy, had at least two documented left ventricular EF measurements (one at or near HAART initiation or during early treatment and one at the most recent available follow-up), and had sufficient documentation of antiretroviral therapy. The HAART initiation date was determined from electronic medication records, although adherence and regimen-specific exposure could not be consistently verified due to incomplete reporting. Patients were excluded if they were younger than 18 years, lacked documented information on HIV treatment (including unclear HAART status), or lacked adequate follow-up data, such as the absence of a second EF measurement. Of the 125 patients initially identified, 50 met all the inclusion criteria, while the remaining 75 were excluded, predominantly due to missing treatment data or unavailable follow-up echocardiography.

Patients were categorized into three groups based on EF change over time: improved EF, no change in EF, and decreased EF. An EF change of less than 10% was considered clinically insignificant, consistent with recognized interobserver variability in echocardiographic EF measurement. The mean interval between EF measurements was 3.1 years, though the timing of follow-up imaging varied across patients and reflected routine clinical practice rather than standardized intervals. Because of substantial missing covariate data, sensitivity analyses were not feasible, and EF categorization was not supplemented with regression modeling or continuous EF analysis.

The primary outcome was the change in EF over time. Secondary variables included age, sex, ethnicity, alcohol use, and history of chronic kidney disease (CKD). Alcohol use and CKD were identified through clinical documentation and problem-list entries; the severity of these conditions could not be consistently determined. Statistical analyses were performed using IBM SPSS Statistics for Windows, Version 22.0 (Released 2013; IBM Corp., Armonk, NY, USA). Continuous variables were analyzed using Student’s t-test, and categorical variables were compared using chi-square testing, with statistical significance defined as p < 0.05.

## Results

A total of 125 patients were initially considered for the study, with 50 included in the final analysis. The remaining 75 were excluded due to the unavailability of key clinical data, most commonly missing follow-up echocardiographic measurements, incomplete antiretroviral therapy documentation, or insufficient longitudinal clinical records. Because this information was missing, it was not possible to determine whether excluded patients differed significantly from included patients with respect to age, baseline EF, or comorbidities (Table 1).

**Table 1:** Baseline demographic and clinical characteristics of the study population. This table summarizes patient demographics, including age, sex, ethnicity, and key clinical variables among the 50 HIV-infected patients with cardiomyopathy included in the analysis. Distributions of ejection fraction (EF) change categories, alcohol use, and history of chronic kidney disease (CKD) are presented as absolute numbers and percentages.

Demographics	Category	Numbers	Percentage
Number of patients		50	100%
Gender	Male	35	70%
	Female	15	30%
Age	<50 years	17	34%
	>50 years	33	66%
Ethnicity	African Americans	38	76%
	Caucasians	12	24%
	Hispanics	0	0%
	Others	0	0%
Ejection fraction (EF)	Decreased	22	44%
	No change	14	28%
	Increased	14	28%
Alcohol use		29	52.72%
Chronic kidney disease (CKD) history		21	38.18%

Among the 50 patients analyzed, 44% (n = 22) experienced a decrease in EF, 28% (n = 14) had an increase in EF, and 28% (n = 14) showed no change (Table 2). The mean age in the decreased EF group was 55.5 ± 10.3 years, in the no-change group 54.1 ± 10.3 years, and in the increased EF group 47.9 ± 10.5 years. One-way ANOVA was used to compare age across EF categories after confirming normality and homogeneity of variance, demonstrating a statistically significant difference (p = 0.04). When the decreased and no-change EF groups were combined and compared with the increased EF group using a two-sample t-test, the difference remained significant (p = 0.03; 95% CI: 0.9-14.7 years).

**Table 2:** Comparison of demographic and clinical characteristics across ejection fraction (EF) change groups. This table presents patient characteristics stratified by EF trajectory (decreased, no change, and increased), including age, sex, race, alcohol use, and history of chronic kidney disease (CKD). Data are shown as mean ± standard deviation or number (percentage), with p-values reflecting comparisons across EF groups.

Parameters	EF decreased	No change in EF	EF increased	p-value
Numbers	22	14	14	-
Mean age (± SD)	55.5 ± 10.3	54.1 ± 10.3	47.9 ± 10.5	-
Race (African American, %)	77.30% (n = 17)	63.40% (n = 9)	85.70% (n = 12)	0.36
Sex (% of male)	59.00% (n = 13)	78.60% (n = 11)	78.60% (n = 11)	0.32
History of alcohol use (%)	54.50% (n = 12)	57.10% (n = 8)	64.30% (n = 9)	0.84
History of CKD (%)	68.20% (n = 15)	50.00% (n = 7)	50.00% (n = 7)	0.43

Baseline EF values, when available, were similar across EF change categories, although incomplete documentation prevented assessment of continuous EF trajectories. Mean follow-up EF reflected the categorical EF groupings by definition. The interval between baseline and follow-up echocardiograms varied widely (mean 3.1 years, range 1-8 years), which may have contributed to variability in measured EF change.

**Figure 1:**
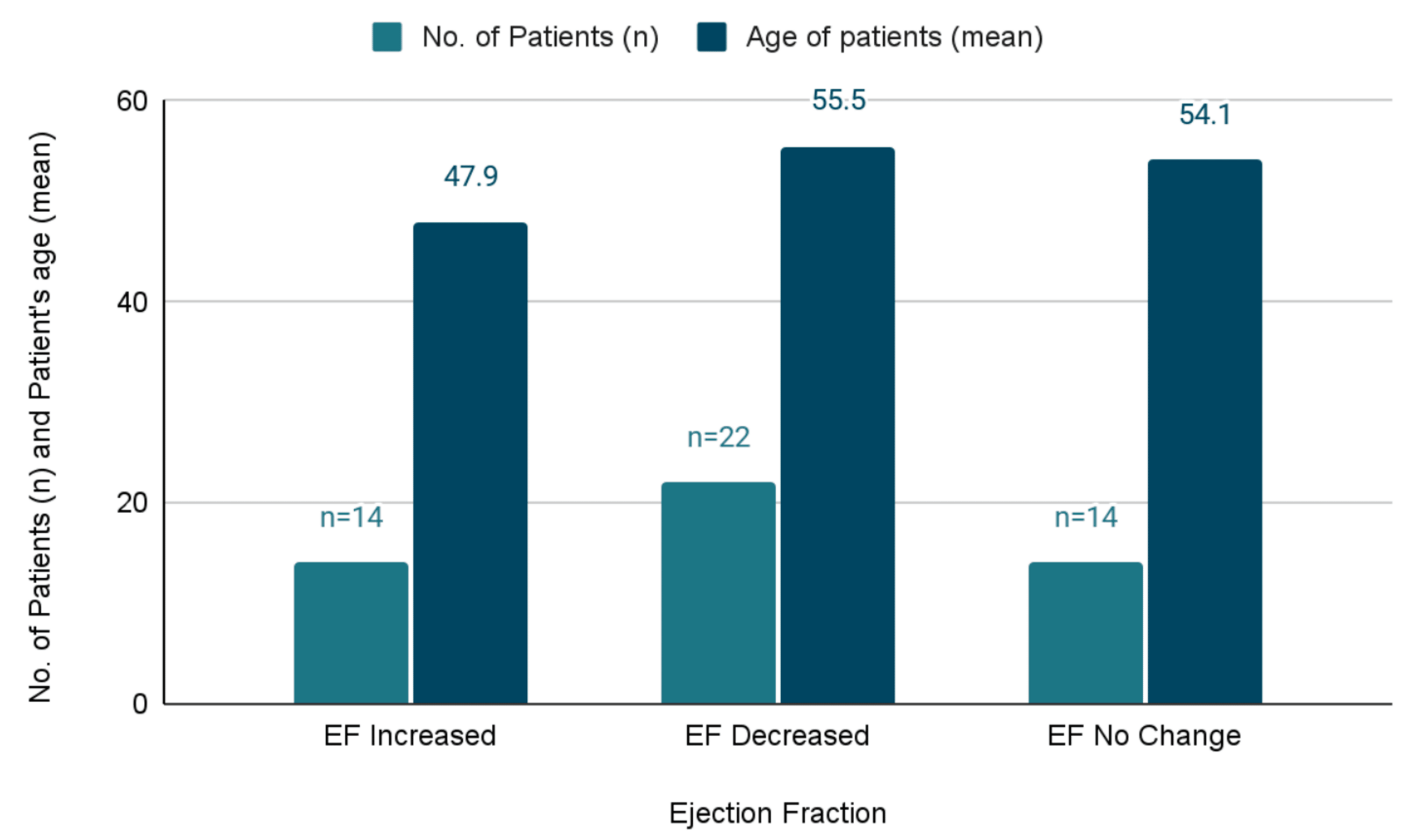
Distribution of patient count and mean age across ejection fraction (EF) change groups. Patients with decreased or unchanged EF were older on average compared with those whose EF improved, highlighting an association between age and EF change during HAART therapy.

Figure 1 illustrates the distribution of patient counts and mean age across EF groups; patients in the decreased and no-change EF categories were older on average than those in the increased EF group, consistent with the statistical findings.

Gender distribution showed that males comprised 59% of the decreased EF group and 78.6% of both the no-change and increased EF groups, although this difference was not statistically significant (p = 0.32). Ethnic distribution demonstrated that African American individuals represented 77.3% of the decreased EF group, 63.4% of the no-change group, and 85.7% of the increased EF group (p = 0.36). Alcohol use was documented in 54.5%, 57.1%, and 64.3% of the three groups, respectively (p = 0.84), and was based on clinical documentation rather than standardized assessment of severity or duration. CKD was present in 68.2% of patients with decreased EF, 50% of those with no change, and 50% of those with increased EF (p = 0.43), with CKD status determined from problem-list entries and physician documentation.

Because follow-up echocardiographic data and detailed antiretroviral exposure profiles were not available for excluded patients, it was not possible to determine whether systematic differences existed between included and excluded individuals. Potential confounding from duration of HIV infection, cumulative HAART exposure, and traditional cardiovascular risk factors could not be assessed due to incomplete data.

Overall, these results demonstrate a significant association between age and EF trajectory among HIV-infected patients with cardiomyopathy receiving HAART. Other demographic and clinical factors, including sex, ethnicity, alcohol use, and CKD, did not differ significantly across EF groups.

## Discussion

In this retrospective cohort study of HIV-infected patients with cardiomyopathy receiving HAART, increasing age was significantly associated with adverse changes in left ventricular EF. Older patients were more likely to experience EF decline or lack of improvement compared with younger individuals. However, this association should be interpreted cautiously, because age may reflect both biological myocardial aging and non-biological factors such as cumulative comorbidity burden, longer duration of HIV infection, prolonged exposure to HAART, or the presence of traditional cardiovascular risk factors not captured in this dataset. Therefore, age should be viewed not only as a biological modifier but also as a surrogate marker for unmeasured clinical variables that may influence EF trajectory.

These findings are consistent with prior studies demonstrating that aging individuals with HIV experience higher rates of cardiovascular disease despite effective antiretroviral therapy [17,18]. Persistent immune activation, chronic inflammation, and cumulative exposure to antiretroviral drugs have been associated with myocardial fibrosis and ventricular dysfunction in several cohorts [12,13,19,20]. Nevertheless, it is important to avoid inferring direct cardioprotective or cardiotoxic effects of HAART from our data, as this study was not designed to isolate medication-specific effects or differentiate outcomes based on regimen class (e.g., protease inhibitors vs. integrase inhibitors). Although antiretroviral therapy has been associated with improvements in systolic function in some younger populations, the long-term effects on remodeling in older adults remain unclear.

Sex and race were not statistically significant predictors of EF change in this analysis. African American males were numerically overrepresented across all EF categories, aligning with broader epidemiologic patterns showing disproportionate burdens of HIV infection and cardiovascular disease in this demographic group [14,15]. However, these findings should be interpreted as hypothesis-generating only, as the study was underpowered to detect subgroup differences.

Several limitations merit consideration. First, the retrospective design limits causal inference. Second, the absence of a non-HAART control group prevents the determination of whether EF changes were related to antiretroviral exposure, underlying cardiomyopathy progression, or unrelated factors. Third, a substantial proportion of initially identified patients were excluded due to missing HAART treatment data or lack of follow-up echocardiography, introducing potential selection and survivorship bias. Patients who did not undergo repeat imaging may have differed systematically in age, comorbidity burden, HIV severity, or clinical stability.

Fourth, traditional cardiovascular risk factors, such as hypertension, diabetes, dyslipidemia, and smoking, were not consistently documented and could not be analyzed. These factors may partially explain the association between age and worsening EF. Fifth, heterogeneity in HAART regimen class, duration of exposure, medication adherence, and degree of viral suppression could not be fully evaluated and may have influenced EF trajectories. Sixth, EF assessments were obtained during routine clinical care and not standardized for research purposes, raising the possibility of inter-observer variability.

Finally, baseline EF severity could not be reliably compared across groups because of incomplete documentation. It therefore remains unclear whether baseline systolic dysfunction modified the relationship between age and subsequent EF change. In addition, because cardiomyopathy etiology could not be uniformly determined, the findings apply primarily to systolic dysfunction rather than broader cardiomyopathy phenotypes.

Despite these limitations, this study highlights an important and clinically relevant pattern: older age was associated with less favorable EF trajectories among HIV-infected patients with cardiomyopathy receiving HAART. These observations underscore the need for future prospective studies with standardized echocardiographic assessments, detailed characterization of HAART exposure, rigorous control of cardiovascular comorbidities, and evaluation of whether baseline systolic dysfunction or cardiomyopathy phenotype moderates long-term remodeling outcomes.

## Conclusions

In this retrospective analysis of HIV-infected patients with cardiomyopathy receiving HAART, older age was associated with less favorable changes in left ventricular EF over time. Because the study did not evaluate the timing of HAART initiation relative to cardiomyopathy onset, these findings should not be interpreted as evidence that earlier HAART initiation directly alters cardiac outcomes; instead, they highlight the broader need to consider age and comorbidity burden when assessing cardiac function in individuals living with HIV.

Recommendations regarding routine baseline and longitudinal echocardiographic monitoring are inferential, as standardized imaging intervals were not evaluated in this study. Nevertheless, the variability in EF trajectories observed underscores the value of clinical vigilance, particularly for patients with known cardiovascular risk factors. While race and sex were not statistically significant predictors of EF change, any observed differences between subgroups should be considered hypothesis-generating rather than indicative of confirmed risk. Future research should address the limitations identified in this study by incorporating prospective designs, clearly defined control populations, and a detailed characterization of antiretroviral regimen class, treatment duration, viral suppression, and traditional cardiovascular risk factors. Standardized echocardiographic follow-up, inclusion of advanced imaging modalities such as strain analysis and cardiac magnetic resonance imaging, and evaluation of baseline systolic function and cardiomyopathy phenotype will be essential in clarifying the long-term effects of HAART on myocardial remodeling and cardiac performance.
